# Supplementary material for: Reply to: Pitfalls in using phenanthroline to study the causal relationship between promoter nucleosome acetylation and transcription
Source: Nat Commun. 2022 Jun 29;13:3725. doi: 10.1038/s41467-022-30351-2 (PMC9243053; doi:10.1038/s41467-022-30351-2)
Supplement: Supplementary file 1 — Reporting Summary [file 41467_2022_30351_MOESM1_ESM.pdf]

nature research

Corresponding author(s): LeAnn Howe

Last updated by author(s): April 15, 2022

Reporting Summary

Nature Research wishes to improve the reproducibility of the work that we publish. This form provides structure for consistency and transparency in reporting. For further information on Nature Research policies, see Authors & Referees and the Editorial Policy Checklist .

Statistics

For all statistical analyses, confirm that the following items are present in the figure legend, table legend, main text, or Methods section.

n/a	Confirmed	
☐	☒	The exact sample size (*n*) for each experimental group/condition, given as a discrete number and unit of measurement
☒	☐	A statement on whether measurements were taken from distinct samples or whether the same sample was measured repeatedly
☐	☒	The statistical test(s) used AND whether they are one- or two-sided<br>*Only common tests should be described solely by name; describe more complex techniques in the Methods section.*
☒	☐	A description of all covariates tested
☒	☐	A description of any assumptions or corrections, such as tests of normality and adjustment for multiple comparisons
☒	☐	A full description of the statistical parameters including central tendency (e.g. means) or other basic estimates (e.g. regression coefficient) AND variation (e.g. standard deviation) or associated estimates of uncertainty (e.g. confidence intervals)
☒	☐	For null hypothesis testing, the test statistic (e.g. *F*, *t*, *r*) with confidence intervals, effect sizes, degrees of freedom and *P* value noted<br>*Give P values as exact values whenever suitable.*
☒	☐	For Bayesian analysis, information on the choice of priors and Markov chain Monte Carlo settings
☒	☐	For hierarchical and complex designs, identification of the appropriate level for tests and full reporting of outcomes
☒	☐	Estimates of effect sizes (e.g. Cohen's *d*, Pearson's *r*), indicating how they were calculated

Our web collection on statistics for biologists contains articles on many of the points above.

Software and code

Policy information about availability of computer code

Data collection	Not applicable
Data analysis	Methods<br>FASTQ files were mapped as described in Martin et al., BAM files were filtered for 100-500 bp reads and total read midpoints overlapping nucleosome depleted regions4 or +1 nucleosomes14, were calculated using the Java Genomics Toolkit (https://github.com/timpalpant/java-genomics-toolkit) ngs.IntervalStats script. For DEseq2 analysis2, size factors accounting for sequencing depth and the scaling factors reported in Martin et al.1 were used. For calling NDRs with increased or decreased Epl1 binding, we used lenient cutoffs of a fold change of greater than 1.5 and an adjusted p-value (FDR) of less than 0.1. For NET-seq data15, processed bedGraphs were downloaded from GSM1673641 and GSM1673642. Sense and antisense read counts in specified windows were calculated using Java Genomics Toolkit, summed across replicates, and violin plots made in Prism. For RNAPII serine 5 phosphorylation ChIP-seq5, FASTQ files were downloaded from the SRA (SRR8450263 and SRR8450261) and mapped using BWA. Fragment centres, estimated using an assumed fragment length of 150 bp, were counted from the TSS to +400 bp. These were depth normalized to compare to RNAPII normalized as described in Martin et al. PCA analysis performed on all 5542 genes (TSS+400) using the regularized log transformation and plotPCA functions of DEseq22.

For manuscripts utilizing custom algorithms or software that are central to the research but not yet described in published literature, software must be made available to editors/reviewers. We strongly encourage code deposition in a community repository (e.g. GitHub). See the Nature Research guidelines for submitting code & software for further information.

Data

Policy information about availability of data

All manuscripts must include a data availability statement. This statement should provide the following information, where applicable:

- Accession codes, unique identifiers, or web links for publicly available datasets
- A list of figures that have associated raw data
- A description of any restrictions on data availability

Published datasets analyzed for this article include "GSE110287 [https://www.ncbi.nlm.nih.gov/geo/query/acc.cgi?acc=GSE110287]", "GSE110286 [https://www.ncbi.nlm.nih.gov/geo/query/acc.cgi?acc=GSE110286]", "GSE68484 [https://www.ncbi.nlm.nih.gov/geo/query/acc.cgi?acc=GSE68484]", and "GSE125226 [https://www.ncbi.nlm.nih.gov/geo/query/acc.cgi?acc=GSE125226]".

Field-specific reporting

Please select the one below that is the best fit for your research. If you are not sure, read the appropriate sections before making your selection.

[x] Life sciences [ ] Behavioural & social sciences [ ] Ecological, evolutionary & environmental sciences

For a reference copy of the document with all sections, see nature.com/documents/nr-reporting-summary-flat.pdf

Life sciences study design

All studies must disclose on these points even when the disclosure is negative.

Sample size	Not applicable
Data exclusions	Not applicable
Replication	Not applicable
Randomization	Not applicable
Blinding	Not applicable

Reporting for specific materials, systems and methods

We require information from authors about some types of materials, experimental systems and methods used in many studies. Here, indicate whether each material, system or method listed is relevant to your study. If you are not sure if a list item applies to your research, read the appropriate section before selecting a response.

Materials & experimental systems

n/a	Involved in the study	
[x]	[ ]	Antibodies
[x]	[ ]	Eukaryotic cell lines
[x]	[ ]	Palaeontology
[x]	[ ]	Animals and other organisms
[x]	[ ]	Human research participants
[x]	[ ]	Clinical data

Methods

n/a	Involved in the study	
[x]	[ ]	ChIP-seq
[x]	[ ]	Flow cytometry
[x]	[ ]	MRI-based neuroimaging
